# Computational Insights on the Competing Effects of Nitric Oxide in Regulating Apoptosis

**DOI:** 10.1371/journal.pone.0002249

**Published:** 2008-05-28

**Authors:** Elife Z. Bagci, Yoram Vodovotz, Timothy R. Billiar, Bard Ermentrout, Ivet Bahar

**Affiliations:** 1 Department of Computational Biology, McGowan Institute for Regenerative Medicine, University of Pittsburgh, Pittsburgh, Pennsylvania, United States of America; 2 Department of Biochemistry and Molecular Genetics, McGowan Institute for Regenerative Medicine, University of Pittsburgh, Pittsburgh, Pennsylvania, United States of America; 3 Department of Surgery, School of Medicine, McGowan Institute for Regenerative Medicine, University of Pittsburgh, Pittsburgh, Pennsylvania, United States of America; 4 Center for Inflammation and Regenerative Modeling, McGowan Institute for Regenerative Medicine, University of Pittsburgh, Pittsburgh, Pennsylvania, United States of America; 5 Department of Mathematics, Arts & Sciences, University of Pittsburgh, Pittsburgh, Pennsylvania, United States of America; University of Bremen, Germany

## Abstract

Despite the establishment of the important role of nitric oxide (NO) on apoptosis, a molecular- level understanding of the origin of its dichotomous pro- and anti-apoptotic effects has been elusive. We propose a new mathematical model for simulating the effects of nitric oxide (NO) on apoptosis. The new model integrates mitochondria-dependent apoptotic pathways with NO-related reactions, to gain insights into the regulatory effect of the reactive NO species N_2_O_3_, non-heme iron nitrosyl species (FeL_n_NO), and peroxynitrite (ONOO^−^). The biochemical pathways of apoptosis coupled with NO-related reactions are described by ordinary differential equations using mass-action kinetics. In the absence of NO, the model predicts either cell survival or apoptosis (a bistable behavior) with shifts in the onset time of apoptotic response depending on the strength of extracellular stimuli. Computations demonstrate that the relative concentrations of anti- and pro-apoptotic reactive NO species, and their interplay with glutathione, determine the net anti- or pro-apoptotic effects at long time points. Interestingly, transient effects on apoptosis are also observed in these simulations, the duration of which may reach up to hours, despite the eventual convergence to an anti-apoptotic state. Our computations point to the importance of precise timing of NO production and external stimulation in determining the eventual pro- or anti-apoptotic role of NO.

## Introduction

The survival of an organism depends on homeostatic mechanisms that establish a balance between cell proliferation and cell death. Apoptosis, a form of programmed cell death, assists in regulating cell proliferation. This process stands in contrast to necrosis, which is thought to be uncontrolled. Dysregulation of apoptosis has been implicated in various disease processes in which the cells apoptose to a higher or lower extent compared to those in healthy tissues [1]. When cells undergo apoptosis, a series of morphological and biochemical changes occur, the mechanisms of which are current topics of broad interest [2].

Apoptosis may be induced by various events, such as binding of extracellular (EC) death signaling ligands to host cell receptors, the lack of pro-survival signals, and genetic damage. These events are usually followed by the activation of caspases, **c**ysteine-dependent **asp**artate-specific prote**ase**s, which initiate and execute apoptosis. Caspases are activated through two major pathways: (a) ligand-dependent or receptor-induced activation (extrinsic pathway), involving death receptors such as Fas or the members of tumor necrosis factor (TNF) receptor superfamily, and (b) mitochondria-dependent activation (intrinsic pathway) via cytochrome *c* (cyt *c*) release from mitochondria, triggered by stress, irradiation or inflammation [3], [4].

Binding of death ligands such as Fas ligand (FasL), TNF, or tumor necrosis-related apoptosis-inducing ligand (TRAIL) usually induces the oligomerization of associated receptors, followed by binding of adaptor proteins, e.g., Fas-Associated Death Domain proteins (FADD), to the cytoplasmic domains of the receptors [5]. The resulting Death Inducing Signaling Complex (DISC) recruits multiple procaspase-8 molecules that mutually cleave and activate one another into caspases-8 (casp8). In Type I cells, large quantities of casp8 activate other caspases including the executioner caspase-3 (casp3) molecules that ultimately lead to apoptosis. In Type II cells, on the other hand, the amount of casp8 activated at the DISC is small, such that the activation of casp8 does not propagate directly to casp3, but instead is amplified via the mitochondria.

Nitric oxide has opposite, competing effects in regulating apoptosis: it exerts an anti-apoptotic effect on hepatocytes [6]–[8], endothelial cells [9]–[13] and keratinocytes [14], whereas it is pro-apoptotic in the case of macrophages [15]–[18]. The variability and complexity of the effects of NO on ultimate cellular fate may arise from this molecule's ability to react with oxygen, reactive oxygen species, metal ions, small thiol-containing molecules, and proteins. The resulting reactive NO species can either trigger or suppress apoptosis through various mechanisms. Chief among them is the S-nitrosative suppression of caspase activation, subsequent to the generation of FeL_n_NO or other species capable of carrying out S-nitrosation reactions (see below) [7], [19]. Differences in the levels of NO and its reaction products may also arise from diverse inflammatory settings in which the expression of nitric oxide synthases (NOS) is affected. For example, quiescent endothelial cells express constitutive NOS (eNOS) that directly produce NO molecules and mediate so-called “direct” effects [20]. Some inflammatory stimuli, on the other hand, lead to inducible NOS (iNOS) expression that subsequently generates reactive NO species, which in turn mediate “indirect” effects of NO. The simultaneous presence of oxygen radicals can generate other reactive NO species that mediate further indirect effects of NO [20]. As another example, hepatocytes and macrophages have different amounts of non-heme iron complexes, which affect the levels of iron-nitrosyl species when NO is produced [21]. Finally, different intracellular levels of glutathione (GSH) can also modulate the time evolution of NO-related compounds [22].

Computational approaches have been used previously to help unravel the complex biology of NO. Biotransport of NO was first modeled by Lancaster [23], [24] followed by other groups, among them Zhang and Edwards [25] (reviewed by Buerk [26]). Recently, Hu and coworkers focused on detailed reaction mechanism of NO [22]. These models have shed light into the biotransport of NO and the types of chemical reactions that involve NO and related reactive species. Additionally, a number of mathematical models have been proposed for understanding the mechanisms of apoptosis [27]–[35], including in particular the work of Eissing et al., which demonstrated the importance of IAP inhibition for imparting bistability in type I cells [30], and that of Rehm et al. [33] and Legewie et al. [32] that showed the same effect in type II cells. These studies have improved our understanding of the robustness of switch mechanisms for regulating apoptosis, but none of them has addressed the dichotomous effects of NO [27]–[35].

Herein, we propose a mathematical model that may shed light on the pro- and anti-apoptotic effects of NO in specific contexts. The model we propose couples the apoptotic cascade [28] to an extended model of NO reaction pathways initially proposed by Hu et al. [22]. First, we illustrate how identical cells can undergo apoptosis at different time points after being exposed to apoptotic stimuli, in accord with experimental data collected on single cells [36], [37]. Then, we examine the apoptotic behavior in response to changes in N_2_O_3_, FeL_n_NO, ONOO^−^, and GSH levels in the presence of NO production by iNOS. Our simulations provide insights into the origin of the dichotomous effects of NO on apoptosis observed in experiments.

## Results

**Figure 1:**
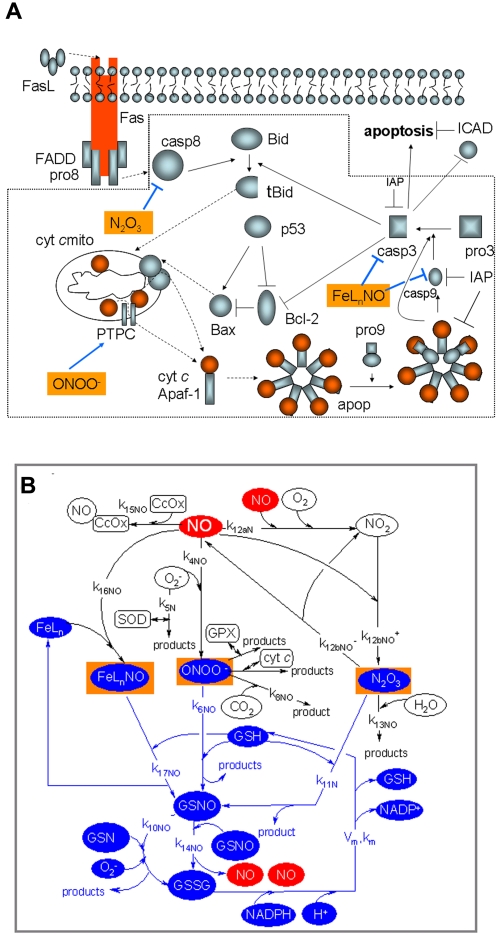
(A) Mitochondria-dependent apoptotic pathways in Model I. The dotted box includes the interactions considered in the model. Solid arrows indicate chemical reactions or upregulation; those terminated by a bar indicate inhibition or downregulation; and dashed arrows indicate subcellular translocation. The components of the model are procaspase-8 (pro8), procaspase-3 (pro3), procaspase-9 (pro9), caspase-8 (casp8), caspase-9 (casp9), caspase-3 (casp3), IAP (inhibitor of apoptosis), cytochrome *c* (cyt *c*), Apaf-1, the heptameric apoptosome complex (apop), the mitochondrial permeability transition pore complex (PTPC), p53, Bcl-2, Bax, Bid, truncated Bid (tBid). The reader is referred to our previous work [28] for more details. Three compounds (N_2_O_3_, FeL_n_NO and ONOO^−^) not included in the original Model I [28] are highlighted. These compounds establish the connection with the nitric oxide pathways delineated in panel B. (B) Nitric oxide (NO)-related reactions in Model II. The following compounds are included: ONOO^−^ (peroxynitrite), GPX (glutathione peroxidase), O_2_^−^ (superoxide), GSH (glutathione), GSNO (nitrosoglutathione), GSSG (glutathione disulfide), C*c*OX (cytochrome *c* oxidase), SOD (superoxide dismutase), FeL_n_ (non-heme iron compounds), FeL_n_NO (non-heme iron nitrosyl compounds), NADPH (reduced form of nicotinamide adenine dinucleotide phosphate), NADP+ (oxidized form of nicotinamide adenine dinucleotide phosphate). FeL_n_NO, ONOO^−^ and N_2_O_3_, highlighted in both panels A and B, bridge between Models I to II. Model III integrates both sets of reactions/pathways through these compounds. GSH modulates their concentrations by reacting with them. GSH is converted by these reactions to GSNO, which is then converted to GSSG and finally back to GSH. Those compounds and interactions are shown in blue. See Table 1 for the complete list of reactions and rate constants.

First, we illustrate how different strengths of EC pro-apoptotic signals may result in opposite qualitative responses or different quantitative (time-dependent) responses in the same type of cells [37], using our recently introduced bistable model [28] (illustrated in Figure 1A). Then, we examine the differences in the bistable response of diverse NO producing cells, e.g. cells with different concentrations of GSH and FeL_n_-and in different settings, i.e., with or without production of superoxide.

**Table 1:** Reactions in Model II

Description of the reaction/interaction	Rate constant (*)	Reference	Reaction index
Production of NO	k_1NO_ = 1 µM/s	[*22*]	*(i)*
Production of O_2_^−^	k_2NO_ = 0.1 µM/s	[*22*]	*(ii)*
Production of GSH	k_3NO_ = 0	[*22*]	*(iii)*
NO+O_2_^−^→ONOO^−^	k_4NO_ = 6700 µM^−1^s^−1^	[*56*]	*(iv)*
SOD+O_2_^−^+H^+^→SOD+½ O_2_+½ H_2_O_2_	k_5NO_ = 2400 µM^−1^s^−1^	[*57*]	*(v)*
ONOO^−^+GSH→GSNO+products	k_6NO_ = 0.00135 µM^−1^s^−1^	[*58*]	*(vi)*
ONOO^−^+GPX→GPX+products	k_7NO_ = 2 µM^−1^s^−1^	[*59*]	*(vii)*
ONOO^−^+CO_2_→products	k_8NO_ = 0.058 µM^−1^s^−1^	[*60]*, [*61*]	*(viii)*
ONOO^−^+cyt *c*→cyt *c*+products	k_9NO_ = 0.025 µM^−1^s^−1^	[*62*]	*(ix)*
2GSNO+O_2_^−^+H_2_O→GSSG+products	k_10NO_ = 0.0006 µM^−2^s^−1^	[*63*]	*(x)*
N_2_O_3_+GSH→GSNO+NO_2_^−^+H^+^	k_11NO_ = 66 µM^−1^s^−1^	[*64*]	*(xi)*
2NO+O_2_→2NO_2_	k_12aNO_ = 0.000006 µM^−2^s^−1^	[*65*]	*(xii)*
NO_2_+NO ↔ N_2_O_3_	k_12bNO_^+^ = 1100 µM^−1^s^−1^	[*65*]	*(xiii)*
	k_12bNO_^−^ = 81000 s^−1^		
N_2_O_3_+H_2_O→products	k_13NO_ = 1600 s^−1^	[*65]*, [66**]	*(xiv)*
GSSG+NADPH+H^+^→2GSH+NADP^+^	V_m_ = 320 µMs^−1^	[*67*]	*(xv)*
	K_m_ = 50 µM		
Cu^+^ GSNO→½ GSSG+NO	k_14NO_ = 0.0002 s^−1^	[*68]*, [*69*]	*(xvi)*
CcOx+NO→CcOX.NO	k_15NO_ = 100 µM^−1^s^−1^	[*70*]	*(xvii)*
FeL_n_+NO→FeL_n_NO	k_16NO_ = 1.21 µM^−1^s^−1^	[*71*]	*(xviii)*
FeL_n_NO+GSH→GSNO+FeL_n_	k_17NO_ = 66 µM^−1^s^−1^ a	[*72*]	*(xix)*
GSH+O_2_^−^→½ GSSG+products	k_17bNO_ = 0.0002 µM^−1^s^−1^	[*73*]	*(xx)*

a Same as k_11NO_

### Delay in apoptosis induction (Model I)

**Figure 2:**
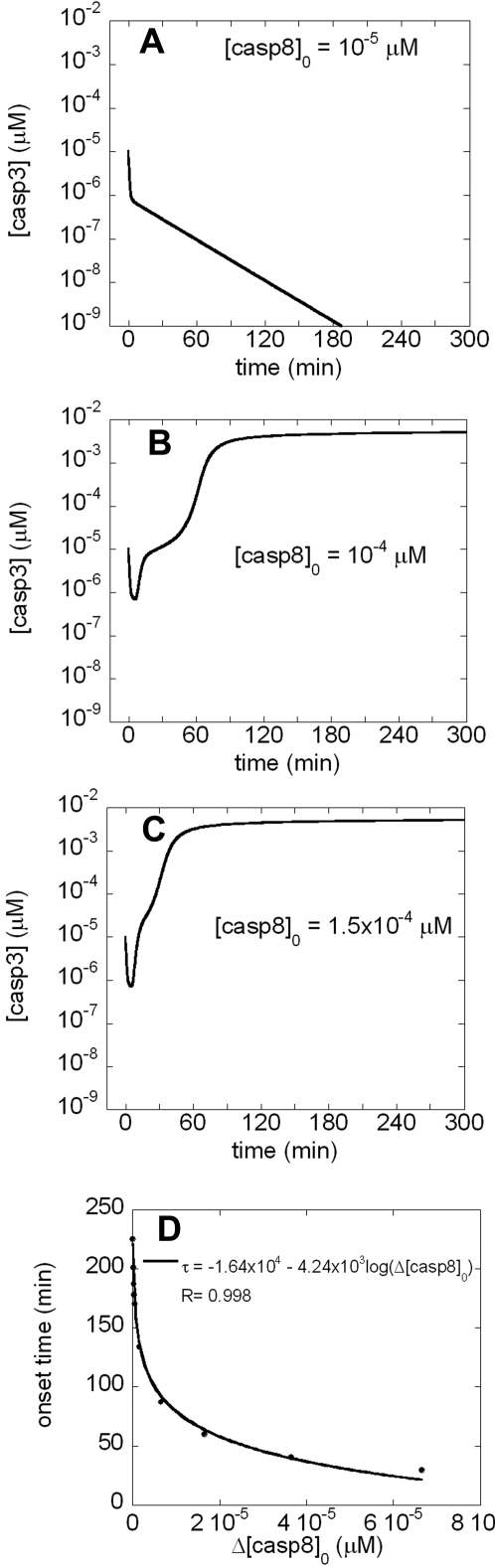
Time evolution of [casp3] predicted by a bistable model in response to different strengths of apoptotic stimuli, A) in a cell subjected to a weak EC apoptotic signal (reflected by the low concentration [caps8]_0_); B) in a cell that is subjected to a stronger EC pro-apoptotic signal. Caspase-3 is activated at 60 minutes; C) in a cell that is subjected to a stronger EC pro-apoptotic signal than one in panel B. Caspase-3 is activated at 30 minutes. Panels A and B illustrate two opposite effects induced by different initial concentrations of caspase-8. The threshold concentration of [caps8]_0_ required for the switch from anti-apoptotic to pro-apoptotic response is calculated to be 8.35×10^−5^ µM. Panels B and C illustrate the shift in the onset time of apoptosis depending on [casp8]_0_. D) Dependence of apoptotic response time on the initial caspase-8 concentration. The ordinate is the onset time of caspase-3 activation, and the abscissa is the initial concentration of caspase-8 in excess of the threshold concentration required for the initiation of apoptosis (evidenced by increase in [casp3], see panels B–C). The onset time of caspase-3 activation exhibits a logarithmic decrease with Δ[casp8]_0_ ([casp8]_0_–8.35×10^−5^ µM).

Tyas et al. [37] showed that cells of the same type simultaneously subjected to EC stimuli initiate their apoptotic response at different times. Figure 2 panels A–C illustrate the theoretical time evolutions of casp3 in three identical cells subjected to different strengths of EC apoptotic stimuli (represented here by the initial concentration of casp8) in the absence of NO. For these simulations, we used Model I with three different values of [casp8]_0_; 10^−5^ µM, 10^−4^ µM, and 1.5×10^−4^ µM in the respective panels A–C, while [casp3]_0_ was 10^−5^ µM in all three cases. Panel A shows that low [casp8]_0_ leads to the depletion of [casp3], while [casp8]_0_ above a certain threshold (8.35×10^−5^ µM) (panels B and C) lead to increase in [casp3] and thereby onset of cell death. Furthermore, comparison of panels B and C shows that a relatively lower [casp8]_0_ (or weaker EC apoptotic signal) results in a time-delayed initiation of apoptosis, in agreement with the single cell experiments done by Tyas et al. [37]. The sharp increase in [casp3] to its equilibrium level indeed starts about 30 minutes later in panel B, compared to panel C.

Next, we examined how this onset time varies with [casp8]_0_. Figure 2D displays the results. An increase in onset time is predicted with decreasing [casp8]_0_ up to [casp8]_0_ = 8.35×10^−5^ µM, after which no apoptotic effect is observed. The time delay is found to obey a logarithmic decay with increasing Δ[casp8]_0_≡[casp8]_0_–8.35×10^−5^ µM, as indicated by the best fitting curve.

This analysis shows that cells of the same type may undergo apoptosis at different times due to their different EC microenvironments. Hence, the difference in the onset times among cells of the same type in a given cell culture may be explained without recourse to alterations in the underlying network of biochemical reactions [28].

### Nitric oxide-associated network (Model II) (Figure 1B)

**Figure 3:**
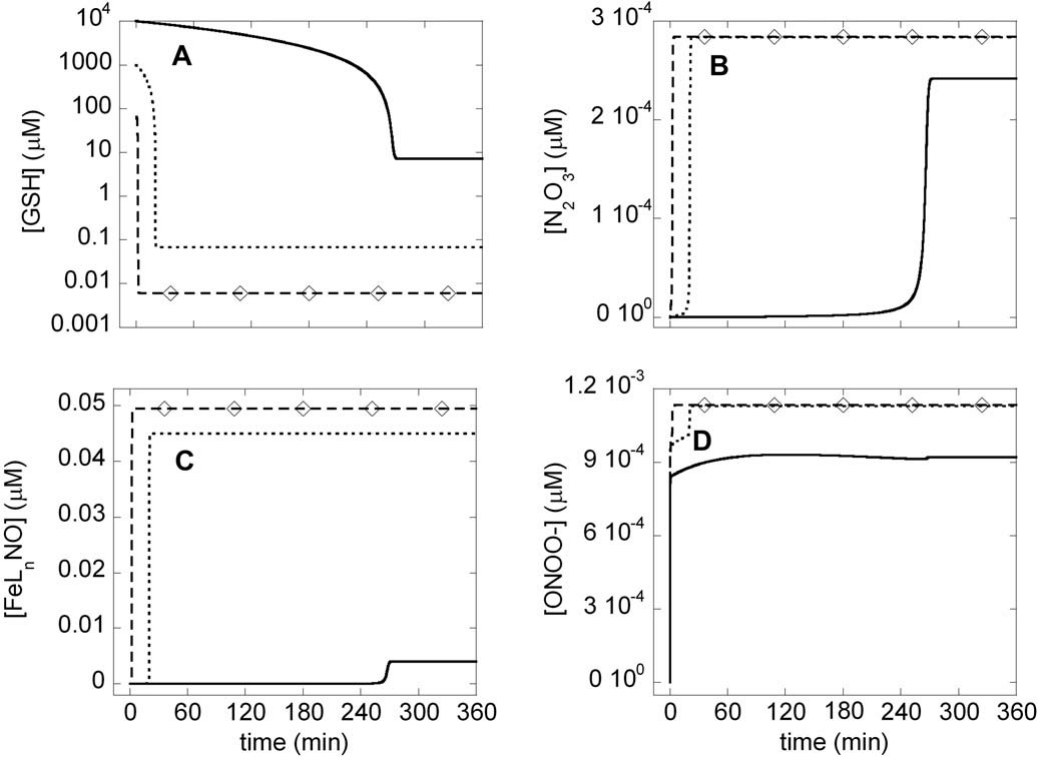
Time evolutions of A) GSH, B) N_2_O_3_, C) FeL_n_NO, and D) ONOO^−^ predicted by Model II. N_2_O_3_ and FeL_n_NO increase to high concentrations by a switch-like mechanism induced by a decrease in GSH concentration due to conversion of GSH to GSNO and subsequently to GSSG. [ONOO^−^] does not follow a similar switch-like increase in its concentration. Solid curve is for [GSH]_0_ = 10^4^ µM, dotted curve for [GSH]_0_ = 10^3^ µM, and dashed curve with diamonds for [GSH]_0_ = 10^2^ µM. The response is thus sharper and earlier in the presence of lower initial concentrations of GSH.

The results from our calculations using Model II are shown in Figure 3. Here, we focused on the time evolution of four compounds, GSH, N_2_O_3_, FeL_n_NO and ONOO^−^, displayed in respective panels A–D. The NO species N_2_O_3_, FeL_n_NO and ONOO^−^ have been proposed to carry out various indirect effects of NO on cellular pathways, including apoptosis, during inflammation [20].

GSH is an anti-oxidant reduced to GSSG by reacting with nitrosative N_2_O_3_ and FeL_n_NO, and with oxidative ONOO^−^ (Table 1). GSH is depleted to low levels in a switch-like manner due to those reactions (panel A). The depletion of GSH is accompanied by increases in N_2_O_3_ and FeL_n_NO concentrations (panels B–C). On the other hand, this switch-like behavior is not that pronounced in [ONOO^−^] time dependence (panel D). Simulations performed with different initial GSH concentrations (three different curves in each panel) change the steady-state concentrations of all three NO-related compounds that interfere with apoptotic pathways (panels B–D). The switch-like increase in [N_2_O_3_] and non-switch-like increase in [ONOO^−^] is in agreement with the results of Hu et al. [22].

### Anti-apoptotic and pro-apoptotic effects of NO (Model III)

We analyze here the dynamics of the reduced mitochondria-dependent apoptosis model coupled to anti- and pro-apoptotic pathways associated with NO; see Materials and Methods for the list of reactions/interactions/steps that come into play in this model (III). As mentioned above, NO-related pathways are coupled to apoptotic pathways through N_2_O_3_, FeL_n_NO, and ONOO^−^ that are produced by the reaction of NO with O_2_, FeL_n_ and O_2_^−^, respectively. For simplicity, those effects of NO mediated by cGMP [38], [39] are not included in this initial mathematical model.

#### Modulating roles of N_2_O_3_ and GSH in apoptosis

**Figure 4:**
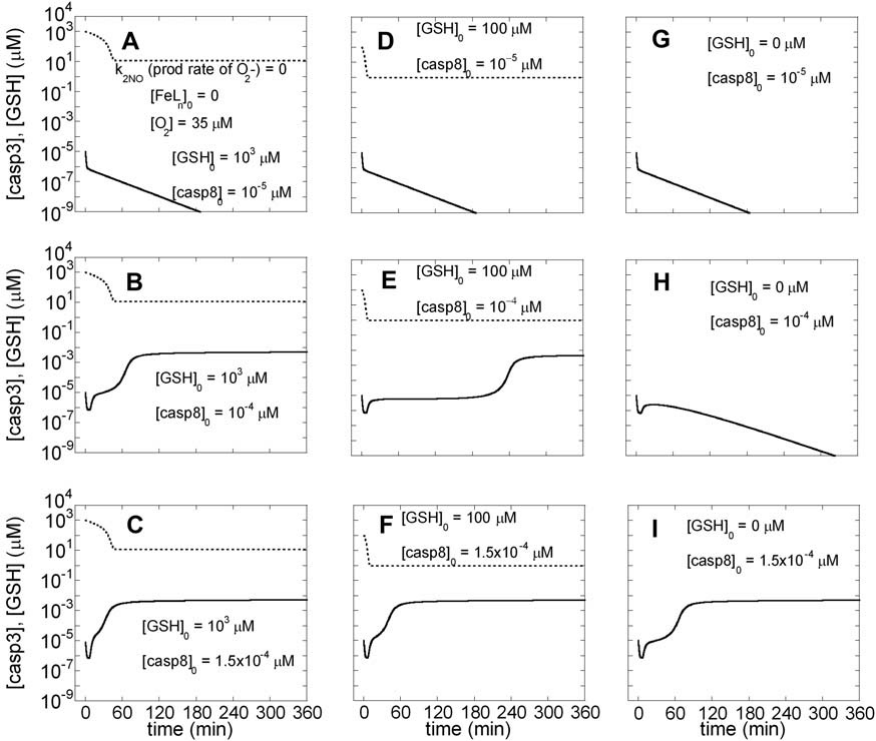
Time evolutions of [GSH] and [casp3] predicted by Model III in the presence of N_2_O_3_ effects. Here, in order to visualize the effect of N_2_O_3_ exclusively, the reaction *(xxii)* in Table 4 is included in the model while those involving FeL_n_NO and ONOO^−^ (reactions (*xx, xxiii-xxv*) are not, assuming FeL_n_ concentration and rate of formation of superoxide to be zero. The solid curves depict the time evolution of [casp3], and dotted curves refer to [GSH]. The three rows of panels are the counterparts of those in Figure 2 A–C, with the different columns referring to different initial concentrations of GSH: A–C) [GSH]_0_ = 10^3^ µM; D–F) [GSH]_0_ = 10^2^ µM; G–I) [GSH]_0_ = 0 µM.

We initially excluded non-heme iron compounds in order to assess the effect of N_2_O_3_ exclusively. The production rate of superoxide was likewise assumed to be zero. N_2_O_3_ is produced by reactions *(xii)* and *(xiii)* in Table 1. NO production and EC stimulation were initiated simultaneously. Figures 4A–C are the counterparts of Figures 2A–C, respectively (same initial conditions, except for the interference of NO pathways through N_2_O_3_), where the time-dependence of [casp3] (solid curve) and [GSH] are shown. The bistable response to apoptotic stimuli, dependent on [casp8]_0,_ is shown to be maintained despite the interference of NO pathways through N_2_O_3_. The three columns refer to different initial concentrations of GSH, decreasing from [GSH]_0_ = 10^3^ (Panels A–C), to [GSH]_0_ = 10^2^ (panels D–F) and GSH]_0_ = 0 (panels G–I). The threshold [casp8]_0_ value for casp3 activation was 8.35×10^−5^ µM in Figure 2, where NO was not produced at all. This value remains the same for both [GSH]_0_ = 10^4^ µM (not shown) and 10^3^ µM (panels A–C) in the presence of NO, but increases to 9.9 ×10^−5^ µM when [GSH]_0_ is 10^2^ µM (panels D–F) and to 1.26 ×10^−4^ µM when [GSH]_0_ is zero (panels G–I), hence the different (pro-apoptotic) behavior observed in panel H.

These results suggest that N_2_O_3_ does not affect the bistable character of the response to EC stimuli, except for modifying the threshold for onset of apoptosis, which is shifted to higher [casp8]_0_ (i.e. rendered more difficult) with decreasing [GSH]_0_. However, high initial concentrations of GSH restore the threshold back to 8.35×10^−5^ µM. Therefore, N_2_O_3_ can serve as an effective modulator of apoptosis provided that the level of GSH in the system is sufficiently low.

#### Effect of N_2_O_3_ on the threshold degradation rates of Bax for transition from bistable to monostable behavior

In our previous computational study of apoptotic pathways, we observed a bistable behavior (selecting between cell death and survival) for degradation rates of Bax (μ_Bax_) lower than a threshold value (0.11 s^−1^), while monostable cell survival was predicted when μ_Bax_>0.11 s^−1^ (Figure 4A in Ref. [28]). This critical value of μ_Bax_ for the transition from bistability to monostability is called a limit point. We explored how the inclusion of NO reactions affects these findings. The limit point value of the Bax degradation rate for monostable cell survival is found to remain unchanged (at 0.11 s^−1^) for the range 10^3^≤[GSH]_0_≤10^4^ µM. However, it decreases to 0.098 s^−1^ for [GSH]_0_ = 10^2^ µM and 0.096 s^−1^ for [GSH]_0_ = 0 µM in the present model. The model again predicts that N_2_O_3_ is not influential when the GSH level is sufficiently high in the cell.

#### Roles of non-heme iron complexes and GSH in apoptotic response

One of the important anti-apoptotic effects of NO is presumed to occur via its ability to react with non-heme iron complexes (FeL_n_) to form FeL_n_NO. These species inhibit caspases by S-nitrosating the catalytic cysteine in the active site of these enzymes [19], [40], [41].

**Figure 5:**
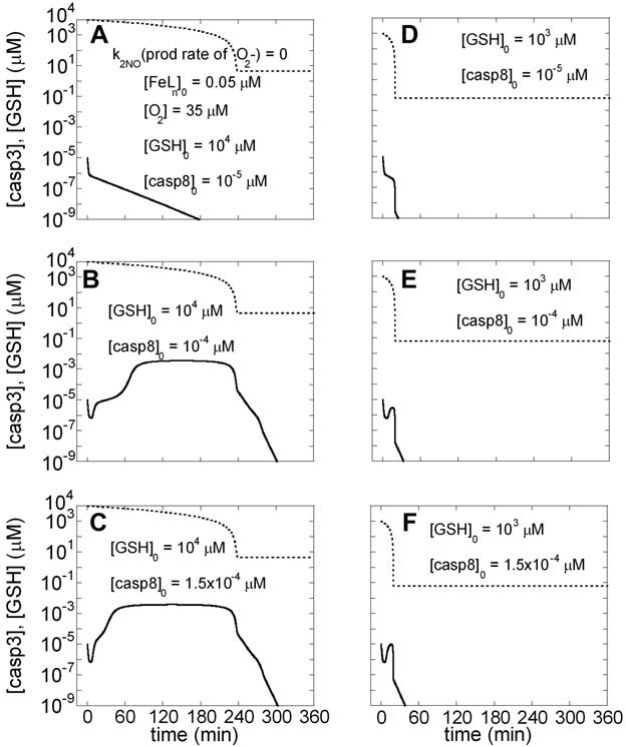
Time evolutions of [GSH] and [casp3] predicted by Model III in the presence of N_2_O_3_ and FeL_n_NO. N_2_O_3_ is present in the model ([O_2_] is non-zero) as well as FeL_n_NO ([FeL_n_]_0_ is non-zero). Each column is a counterpart of Figure 2A–C with different initial concentrations of GSH. A–C) [GSH]_0_ = 10^4^ µM; D–F) [GSH]_0_ = 10^3^ µM. Solid curve shows the time evolution of [casp3] and dotted curve that of [GSH].

The results are presented in Figure 5, panels A-F, organized similarly to Figure 4 (i.e. using different [casp8]_0_ in each row, and different [GSH]_0_ in the two columns). Our calculations suggest that when the FeL_n_ concentration is higher than 0.03 µM, there are no longer two stable steady-states at long times: caspase-3 levels always decrease to zero, even though their time evolutions depend on [casp8]_0_ and [GSH]_0_. Yet, depending on the level of GSH, both apoptosis and cell survival may be possible. Panels A–C correspond to relatively high [GSH]_0._ In panel A, [casp3] decreases to 10^−8^ µM that is less than 1 molecule per cell, hence zero, from 10^−5^ µM within the first two hours. However, in panels B and C, [casp3] increases to nanomolar values and remains at those levels for more than three hours. Caspase-3 may cause enough damage to kill the cell before it is depleted at longer times. We note that lower [GSH]_0_ (e.g. [GSH]_0_ = 10^3^ µM, panels D–F and [GSH]_0_<10^3^ µM, data not shown) do not permit the casp3 concentration to reach such pro-apoptotic levels and monostable cell survival is observed irrespective of [casp8]_0_.

Various cell types subject to different intracellular microenvironments, or even the same cells under different settings (e.g. healthy state vs. inflammation or oxidative stress), may produce or experience different reactive NO intermediates [7], [20], [42]. For example, more FeL_n_NO may be produced in hepatocytes than in RAW264.7 macrophage-like cells due to the high level of non-heme iron complexes in hepatocytes [21]. In our previous study, RAW264.7 cells underwent apoptosis in the presence of NO; conversely, no casp3 activation was observed in either hepatocytes or iron loaded RAW264.7 cells [21]. The results (Figure 4 and data not shown) suggest that in cells with iron concentrations lower than 0.03 µM (e.g. RAW264.7 cells), both cell survival and apoptosis are possible depending on the strength of apoptotic stimuli (in agreement with our experimental results) [21]. However, a change in the intracellular environment of the same cell can change the response. Figure 5D–F shows that casp3 is not activated in the presence of non-heme iron ([FeL_n_]_0_ = 0.05 µM) when [GSH]_0_ = 10^3^ µM and [GSH]_0_<10^3^ µM (data not shown). We also checked if casp3 is activated when [casp8]_0_ is as high as 0.1 µM when [GSH]  = 10^3^ µM. In this case, caspase-3 concentration increased to 0.0007 µM for approximately 5 minutes, an apoptotic stimulus that is likely insufficient for apoptosis. This prediction is in good agreement with our observation that caspase-3 is not activated in non-heme iron-loaded RAW264.7 cells whose [GSH]_0_ does not reach 10^4^ µM [21].

#### Roles of ONOO^−^ and GSH in apoptotic response

The mechanism by which NO or its reactive species exert pro-apoptotic effects is not well established [43]. In the present study, we assume that the pro-apoptotic effect of NO occurs via formation of ONOO^−^, as has been suggested from a large number of experimental studies both *in vitro* and *in vivo* [44], [45]. Experimental studies suggest that ONOO^−^ may induce the opening of mitochondrial permeability transition pores (MPTPs) and subsequent cyt *c* release from mitochondria [38].

**Figure 6:**
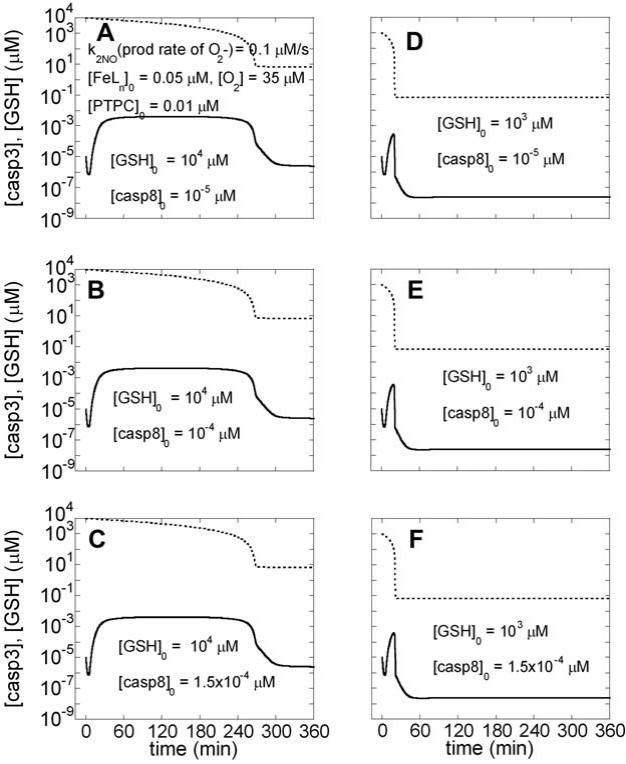
Time evolutions of [GSH] and [casp3] predicted by Model III in the presence of N_2_O_3_, FeL_n_NO and ONOO^−^. The initial concentration of PTPC is 0.01 µM. Each column is a counterpart of Figure 2A–C and has a different initial concentration for GSH. A–C) [GSH]_0_ = 10^4^ µM; D–F) [GSH]_0_ = 10^3^ µM. Solid line is for time evolution of [casp3] and dashed line is for time evolution of [GSH]. Caspase-3 concentrations at long times are 2.4 ×10^−4^ µM and 2.5×10^−8^ µM for panels A–C and D–F, respectively.

The possible mechanisms of cyt *c* release from mitochondria are diverse and controversial [46], [47]. In our model, we assume that cyt *c* release is mediated by activation of MPTPs, independent of Bax channel formation on mitochondria. The complex that forms the MPTPs is called mitochondrial permeability transition pore complex (PTPC). The complex consists of peripheral benzodiazepine receptor, cyclophilin D, adenine nucleotide translocator (ANT), voltage-dependent anion channel (VDAC), and other proteins [48]. ANT is proposed to be converted from a specific transporter to a non-specific pore which then releases cyt *c* into the cytoplasm and subsequently induces apoptosis. It has been suggested that ONOO^−^ acts on PTPC, specifically on ANT, to convert it to a non-specific pore (PTPC_act_) [49]. We represent this process as:
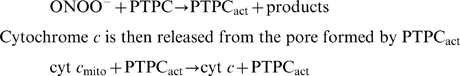



The results are shown in Figure 6. The initial concentration of PTPC is assumed to be high (0.01 µM). At that value, Model I predicts the response to apoptotic stimuli to be monostable apoptosis (Figure 6 in Ref. [28]). We see a similar response in Figure 6A; a low initial value of casp8 (10^−5^ µM) results in an increase of [casp3] to nanomolar levels. Casp3 activation was observed with even lower values of [casp8]_0._ However, casp3 does not reach nanomolar concentrations when [GSH]_0_ = 10^3^ µM (Figures 6D–F) and [GSH]_0_<10^3^ µM (data not shown). Initial concentrations [casp8]_0_ higher than 1.5×10^−4^ µM did not change this prediction.

These results suggest that in cells with large numbers of MPTPs (probably with high numbers of mitochondria), there are two possible outcomes in the presence of NO and O_2_^−^ production: pathological cell death when GSH level is high (10^4^ µM) and solely cell survival when GSH level is low ([GSH]≤10^3^ µM) in the presence of O_2_ and FeL_n_. This result stands in contrast with studies in which GSH protects against oxidative stress (high concentrations of O_2_^−^ and ONOO^−^) that can cause apoptosis. The reason for this paradoxical prediction is that GSH has both protective and pro-apoptotic effects in our simulations: it exerts apoptotic effects via its reaction with anti-apoptotic N_2_O_3_ and FeL_n_, and protective effects due to its reaction with pro-apoptotic O_2_^−^ and ONOO^−^. Simulations (Figure 6) suggest that the pro-apoptotic effect of GSH is stronger than its protective effect using the interactions and parameters adopted in current simulations.

**Figure 7:**
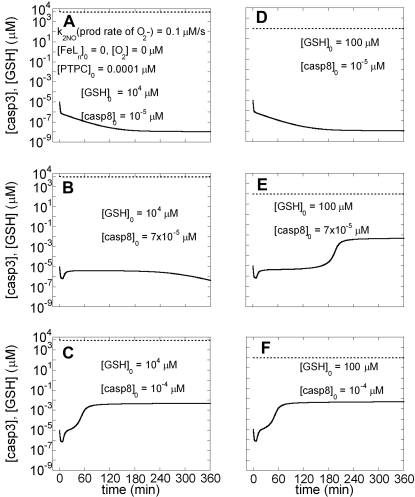
Time evolutions of [GSH] and [casp3] predicted by Model III in the absence of N_2_O_3_, FeL_n_NO and presence of ONOO^−^. The initial concentration of PTPC is 0.0001 µM. A–C) [GSH]_0_ = 10^4^ µM; D–F) [GSH]_0_ = 10^2^ µM. Solid line is for time evolution of [casp3] and dashed line is for time evolution of [GSH].

To examine the possibility of an alternative response, we repeated the computations depicted in Figure 6 in the absence of O_2_ (so that N_2_O_3_ is not produced) and FeL_n_. We also used initial PTPC concentration of 0.0001 µM, at which Model I predicts bistability (Figure 6 in ref [28]). As seen in Figure 7, both cell survival and apoptosis are possible under these conditions, depending on [casp8]_0_. Higher [GSH]_0_ (10^4^ µM) results in cell survival (Figure 7B) in contrast to lower [GSH]_0_ resulting in apoptosis (Figure 7E) under the same amount of EC stimulus ([casp8]_0_ = 7×10^−5^ µM). The present analysis thus shows that the protection by GSH against oxidative stress is possible provided that O_2_ and FeL_n_ levels are sufficiently low.

## Discussion

We present here the results from simulations that incorporate the main chemical interactions of NO with components of the apoptotic interactions network, with the goal of shedding light on the dichotomous effects of NO on apoptosis. Based on previously published studies, we considered N_2_O_3_ and FeL_n_NO to be anti-apoptotic and ONOO^−^ pro-apoptotic. The results predict that cell survival or apoptosis is determined by a complex interplay among these reactive NO species and GSH. We observed that relative concentrations of anti-apoptotic and pro-apoptotic species determine the ultimate cell fate at late time points. Interestingly, transient apoptotic effects were observed under specific conditions (e.g. Figure 5 panels B–C). These intriguing findings point to the importance of the *timing* of NO production and apoptotic stimuli in determining the actual anti- or pro-apoptotic effect, even if steady state conditions favor cell survival, in agreement with our previous observations [50]–[52]. Another interesting effect we observed in our simulations was the time shift/delay in the onset of apoptosis in the presence of weak EC stimulus (panel B–D in Figure 2), consistent with the experiments of Tyas et al. [37].

Our simulations suggest that N_2_O_3_ and non-heme iron nitrosyl form in a switch-like manner after depletion of GSH. ONOO^−^ formation, on the other hand, hardly shows any switch-like behavior. We further found that N_2_O_3_ does not eliminate the bistability between cell survival and apoptosis, but rather increases the threshold [casp8]_0_ for onset of apoptosis. However, high initial concentrations of GSH restore the threshold back to its original value. Therefore, we would predict, non-intuitively, that N_2_O_3_ does not influence cell survival when [GSH]_0_ level is high.

On the other hand, our simulations suggest that there are no longer two stable steady states (cell survival and apoptosis) in the presence of non-heme iron at a level higher than a threshold value. Caspase-3 levels always decrease to zero even though its time evolution may depend on [casp8]_0_ and [GSH]_0_. Yet, despite the steady state conditions that favor cell survival, executioner caspase concentrations can reach and retain apoptotic levels for several hours before they level off, when [GSH]_0_ is high. When [GSH]_0_ is low, on the other hand, our simulations predict resistance to apoptosis, in agreement with experimental observation [21].

In cells with high numbers of MPTPs (probably cells that contain high numbers of mitochondria), our simulations suggest two possibilities in the presence of simultaneous NO and O_2_^−^ production and sufficiently high [FeL_n_]_0_: pathological cell death when [GSH]_0_ is high (10^4^ µM) or solely cell survival when [GSH]_0_ level is low ([GSH]_0_≤10^3^ µM). On the other hand, GSH is protective against oxidative stress when O_2_ and FeL_n_ levels are low in cells with low numbers of MPTPs.

Tiedge et al. [53] have shown that pancreatic beta cells have low anti-oxidant levels (notably, GSH) and that the number of mitochondria is a determining factor in survival. They have also shown that transfection of the cells with a peroxide-inactivating enzyme, catalase, can protect against high-glucose induced apoptosis. An interesting experiment would be to correlate the number of mitochondria in the transfected cells with their survival. Oyadomari et al. [54] have shown that the endoplasmic reticulum (ER) plays a crucial role in the fate of NO-sensitive beta cells via calcium signaling. A natural next step in the present model would be to include these effects via a model which incorporates the effects of NO on the ER.

Our results are subject to several limitations. While we have adopted values for kinetic parameters and concentrations in accord with experimental data whenever available (Tables 1 and 2), many of the true intracellular rate constants for the reactions in our simulations are unknown. Given that the observed apoptotic responses are so sensitive to model parameters, detailed knowledge of reaction mechanisms and accurate values of rate constants are needed in modeling reaction networks as complicated as the ones presented here. Due to an extensive literature basis, we have posited that the pro-apoptotic NO species is ONOO^−^; however, other species may in fact exert this effect. Additionally, the hypotheses raised by our simulations remain to be tested by further experiments. Some of the predictions could be tested by iron chelation and/or treatment with superoxide donors in a cell-free system or in single-cell studies, though each of these manipulations may have additional, artifactual effects. The hypothesis of bistability with regards to the apoptotic response can be tested as suggested by Legewie et al. [32], either in cell free-systems by adding caspase-3 or in single living cells by microinjecting caspase-3. The time evolution of caspase-3 can be monitored by fluorescent caspase-3 substrates. The time needed for caspase-3 activation will increase abruptly as caspase-3 concentration added will approach threshold value in a bistable system (Figure 2D). Such combined experimental and computational studies may potentially help us understand and design therapeutics for diseases associated with apoptosis dysregulation.

**Table 2:** Equilibrium levels and initial concentrations used in Model II

Equilibrium concentrations	References
[SOD]_∞_ = 10 µM	[*74*]
[GPX]_∞_ = 5.8 µM	[*67*]
[CO_2_]_∞_ = 10^3^ µM	[*61*]
[O_2_]_∞_ = 35 µM	[*67*]
[cyt *c*]_∞_ = 400 µM	[*75*]
**Initial concentrations**	**References**
[CcOx]_0_ = 0.1 µM	[*76*]
[FeL_n_]_0_ = 0.05 µM	[*77*]
[GSH]_0_ = 10^4^ µM (or otherwise specified)	[*22*]

## Materials and Methods

### Models

Three models are considered in this study. **Model I**, proposed in our earlier work [28], focuses on the pathways involved in mitochondria-dependent apoptosis (Figure 1A). **Model II** is an extension of the kinetic model of NO-associated reactions recently proposed by Hu et al. [22] (Figure 1B). Finally, **Model III** is the integration of Models I and II, proposed in the present study, to examine the pro-apoptotic and anti-apoptotic effects of NO.

All interactions (chemical or physical; single step or multiple steps) are modeled using mass action kinetics theory and methods. The simulations are performed using XPPAUT software (http://www.math.pitt.edu/bard/xpp/xpp.html) [55].

### Model II-Generation of NO-related oxidative and nitrosative species ONOO^−^, N_2_O_3_, and FeL_n_NO

We extended the network originally proposed by Hu and coworkers [22] by introducing additional reactions involving NO, as well as additional compounds such as the NO-related species FeL_n_NO (L denotes ligands that do not contain heme), NO_2_, and cytochrome *c* oxidase (C*c*Ox). Figure 1B illustrates the extended network of interactions. Table 1 lists the corresponding reactions (indexed as *(i)–(xx)*) and rate constants. The reactions *(xii)* and *(xiii)* break down the production of N_2_O_3_ from NO and O_2_ into two steps that replace the corresponding reaction (with rate constant k_12_) used in the model of Hu et al. [22]. Reactions *(xvi)–(xx)* are introduced in the present study. The identity of the products are not written when these compounds do not serve as reactants in any of the reactions listed in Table 1. Table 3 lists the rate laws for these reactions (the first 20 rows), which are used in the differential rate equations (rows 21–29) that control the time evolution of the concentration of the individual compounds. Model II contains 16 components. Eleven of them reach steady-state concentrations within a short time interval (∼20 minutes) after initiation of the simulations for [GSH]_0_≤10^3^ µM and within four and half hours for [GSH]_0_ = 10^4^ µM, whereas five compounds (superoxide dismutase (SOD), glutathione peroxidase (GPX), CO_2_, O_2_, and cyt *c*) retain their equilibrium concentrations. Table 2 lists the initial and equilibrium concentrations different from zero, adopted in Model II, and the corresponding references.

**Table 3:** Rate equations for Model II (*)

Rate laws (Eq.s 1–20) and differential rate equations (Eq.s 21–29)	Equation numbers
r_1NO_ = k_1NO_	(1)
r_2NO_ = k_2NO_	(2)
r_3NO_ = k_3NO_	(3)
r_4NO_ = k_4NO_[NO][O_2_^−^]	(4)
r_5NO_ = k_5NO_[SOD][O_2_^−^]	(5)
r_6NO_ = k_6NO_[ONOO^−^][GSH]	(6)
r_7NO_ = k_7NO_[ONOO^−^][GPX]	(7)
r_8NO_ = k_8NO_[ONOO^−^][CO_2_]	(8)
r_9NO_ = k_9NO_[ONOO^−^][cyt *c*]	(9)
r_10NO_ = k_10NO_[GSNO]^2^[O_2_^−^]	(10)
r_11NO_ = k_11NO_[N_2_O_3_ ][GSH]	(11)
r_12aNO_ = k_12aNO_[NO]^2^[O_2_]	(12)
r_12bNO_^+^ = k_12bNO_^+^[NO_2_][NO]	(13)
r_12bNO_^−^ = k_12bNO_^−^[N_2_O_3_]	(14)
r_13NO_ = k_13NO_[N_2_O_3_]	(15)
r_m_ = V_m_[GSSG]/(K_m_+[GSSG])	(16)
r_14NO_ = k_14NO_[GSNO]	(17)
r_15NO_ = k_15NO_[CcOx][NO]	(18)
r_16NO_ = k_16NO_[FeL_n_][NO]	(19)
r_17NO_ = k_17NO_[FeL_n_NO][GSH]	(20)
d[NO]/dt = r_1NO_–r_4NO_–2r_12aNO_–r_12bNO_^+^+r_12bNO_^−^+r_14NO_ –r_15NO_ *–*r_16NO_	(21)
d[O_2_^−^]/dt = r_2NO_–r_4NO_–r_5NO_–r_10NO_	(22)
d[ONOO^−^]/dt = r_4NO_–r_6NO_–r_7NO_–r_8NO_–r_9NO_	(23)
d[GSH]/dt = r_3NO_–r_6NO_–r_11NO_+2r_m_ *–*r_17NO_	(24)
d[GSNO]/dt = r_6NO_–2r_10NO_+r_11NO_ –r_14NO_ *+*r_17NO_	(25)
d[N_2_O_3_]/dt = –r_11NO_+r_12bNO_^+^–r_12bNO_^−^–r_13NO_	(26)
d[NO_2_]/dt = 2r_12aNO_–r_12bNO_^+^+r_12bNO_^−^	(27)
d[CcOx]/dt = –r_15NO_	(28)
d[FeL_n_]/dt = –r_16NO_+r_17NO_	(29)

(^*^) Note that [FeL_n_NO] = [FeL_n_]_0_−[FeL_n_], and [GSSG] = ([GSH]_0_–[GSH]–[GSNO])/2

### Model III–Effects of NO-related reactions on apoptotic pathways

Model III combines Models I and II upon inclusion of the additional reactions presented in Table 4. See the highlighted compounds in Figure 1, which point to the species that couple the apoptotic and NO pathways. We note that ONOO^−^ has a pro-apoptotic effect, while N_2_O_3_ and FeL_n_NO (reactions labeled *(xxii)–(xxv)*) deactivate the caspases, thus inducing anti-apoptotic effects. The associated rate constants and references are given in Table 4. Table 5 provides the rate expressions (rows 30–34) and differential rate equations (rows 35–43) for these reactions and involved compounds, respectively.

**Table 4:** Reactions bridging between Models I to II (*)

Reaction	Rate constant	Reference	Reaction index
ONOO^−^+PTPC→PTPC_act_+products	k_18NO_	accounts for ONOO^−^ induced formation of non-specific pore associated with mitochondrial permeability transition [49]	*(xxi)*
N_2_O_3_+casp8→casp8.NO+FeL_n_	k_19NO_	[*78*]	*(xxii)*
FeL_n_NO+casp8→casp8.NO+FeL_n_	k_20NO_	[*38*]	*(xxiii)*
FeL_n_NO+casp9→casp9.NO+FeL_n_	k_21NO_	[*38*]	*(xxiv)*
FeL_n_NO+casp3→casp3.NO+FeL_n_	k_22NO_	[*38*]	*(xxv)*

(^*^) The parameters used in the present study are k_18NO_ = 1 µM^−1^s^−1^ (varying the value between 0.01 µM^−1^s^−1^ and 100 µM^−1^s^−1^ does not affect the results), k_19NO_ = 10 µM^−1^s^−1^ [*78*], k_20NO_ = k_21NO_ = k_22NO_ = 66 µM^−1^s^−1^ (the same value as k_11NO_).

**Table 5:** The modified equations from either Model I or II (*)

Rate laws (Eq.s 30–34) and differential rate equations (Eq.s 35–43)	Equation numbers
r_18NO_ = k_18NO_[ONOO^−^][PTPC]	(30)
r_19NO_ = k_19NO_[N_2_O_3_][casp8]	(31)
r_20NO_ = k_20NO_[FeL_n_NO][casp8]	(32)
r_21NO_ = k_21NO_[FeL_n_NO][casp9]	(33)
r_22NO_ = k_22NO_[FeL_n_NO][casp3]	(34)
d[ONOO^−^]/dt = r_4NO_–r_6NO_–r_7NO_–r_8NO_–r_9NO_–r_18NO_	(35)
d[PTPC]/dt = –r_19NO_	(36)
d[N_2_O_3_]/dt = –r_11NO_+r_12bNO_^+^–r_12bNO_^−^–r_13NO_–r_19NO_	(37)
d[casp8]/dt = −J_0_+J_0_^f^+J_casp8_–r_19NO_–r_20NO_ (*)	(38)
d[FeL_n_NO]/dt = r_16NO_–r_17NO_–r_20NO_–r_21NO_–r_22NO_	(39)
d[FeL_n_]/dt = –r_16NO_+r_17NO_+r_20NO_+r_21NO_+r_22NO_	(40)
d[casp9]/dt = J_4_ –J_4b_–J_5_–J_6_ +J_6_^f^+J_casp9_–r_21NO_ (*)	(41)
d[casp3]/dt = J_6_^f^+J_6b_^f^–J_7_ –J_8_+J_8_^f^–J_9_+J_9_^f^+J_casp3_–r_22NO_ (*)	(42)
d[cyt *c*]/dt = J_14–_J_1_+J_cyt*c*_+k[PTPCact][cyt *c*_mit_] where k = 1 µM^−1^s^−1^ (*)	(43)

(^*^) J refers to fluxes of components, for details see ref [28]. PTPC_act_ refers to the nonspecific pore at the mitochondria that releases cyt *c*. Note that [PTPC_act_] = [PTPC]_0_–[PTPC].

The steady-state concentrations [H^+^]_∞_ in reaction (v), [H_2_O]_∞_ in reactions (x) and (xiv), [NADPH]_∞_ and [H^+^]_∞_ in reaction (xv), [Cu^+^]_∞_ in reaction (xvi) are incorporated into the corresponding rate constants.
